# Specialties in Medicine and the Paris Faculty

**Published:** 1860-04

**Authors:** 


*Specialties in Medicine and the Paris Faculty.*—Early in last year the Minister of Public Instruction addressed the following question to the Paris Faculty of Medicine: "Are the various branches of Medical Science sufficiently represented in the teaching of the Faculty?" This query was evidently but a polite mode employed by the Minister of acquainting the Faculty with his opinion of the insufficiency of its instruction, as also with the liberality of his disposition to provide the means for rendering it complete. He, however, had the courtesy to leave to the Faculty the merit and honor of taking the initiative in the matter. At the end of three months the Faculty replied to his generous provocation, with the following resolution, carried by a large majority: "That the creation of chairs for specialties would be a very disastrous measure, which would alter the proper character of education, and would prove of no utility for the practical instruction of the student." M. Latour, in the *Union Médicale*, observes: "We hope that the liberal intentions of the Minister will not be discouraged by the resistance of the Faculty. History teaches us with what difficulty corporations accept any innovation which disturbs their quietude. It is not a century since the old Paris Faculty would have felt itself dishonored in seeing any of its chairs bestowed upon the surgeons. They now occupy nearly one-half of these. The spirit of progress has dissipated all these contemptible vanities. Let us still trust to it." M. Diday, of Lyons, has published an eloquent protest against the decision of the Paris Faculty, maintaining that it is the duty of the Faculty to furnish a complete course of instruction to its students, which at the present time it does not do. He maintains that specialties have been the source and cause of the greatest scientific advances, and that the very men who are so jealous of allowing them to be taught are only too happy to call in their aid in the difficulties of practice. Even as it is now, the official teaching is much concerned with specialties. Surgery, taken upon its whole, is but a specialty; Operative Surgery is but a specialty of a specialty; and obstetrics, hygiene, legal medicine, pharmacology, materia medica, &c., are one and all but specialties. M. Diday insists, at great length, upon the absolute necessity of founding two new specialties in the Paris Faculty, that of Psychiâtrie and of Syphiliographie.—*Medical Times and Gazette.*

